# The use of remote sensing to derive maize sowing dates for large-scale crop yield simulations

**DOI:** 10.1007/s00484-020-02050-4

**Published:** 2020-11-30

**Authors:** Ehsan Eyshi Rezaei, Gohar Ghazaryan, Javier González, Natalie Cornish, Olena Dubovyk, Stefan Siebert

**Affiliations:** 1 Department of Crop Sciences, University of Göttingen, 37075 Göttingen, Germany; 2 Leibniz Centre for Agricultural Landscape Research (ZALF), Müncheberg, Germany; 3 Center for Remote Sensing of Land Surfaces (ZFL), University of Bonn, 53113 Bonn, Germany; 4 RSS Remote Sensing Solutions GmbH, Dingolfinger Strasse 9, 81673 Munich, Germany

**Keywords:** Crop modeling, Remote sensing, Drought, Maize, Sowing date, South Africa, MODIS

## Abstract

One of the major sources of uncertainty in large-scale crop modeling is the lack of information capturing the spatiotemporal variability of crop sowing dates. Remote sensing can contribute to reducing such uncertainties by providing essential spatial and temporal information to crop models and improving the accuracy of yield predictions. However, little is known about the impacts of the differences in crop sowing dates estimated by using remote sensing (RS) and other established methods, the uncertainties introduced by the thresholds used in these methods, and the sensitivity of simulated crop yields to these uncertainties in crop sowing dates. In the present study, we performed a systematic sensitivity analysis using various scenarios. The LINTUL-5 crop model implemented in the SIMPLACE modeling platform was applied during the period 2001–2016 to simulate maize yields across four provinces in South Africa using previously defined scenarios of sowing dates. As expected, the selected methodology and the selected threshold considerably influenced the estimated sowing dates (up to 51 days) and resulted in differences in the long-term mean maize yield reaching up to 1.7 t ha^−1^ (48% of the mean yield) at the province level. Using RS-derived sowing date estimations resulted in a better representation of the yield variability in space and time since the use of RS information not only relies on precipitation but also captures the impacts of socioeconomic factors on the sowing decision, particularly for smallholder farmers. The model was not able to reproduce the observed yield anomalies in Free State (Pearson correlation coefficient: 0.16 to 0.23) and Mpumalanga (Pearson correlation coefficient: 0.11 to 0.18) in South Africa when using fixed and precipitation rule-based sowing date estimations. Further research with high-resolution climate and soil data and ground-based observations is required to better understand the sources of the uncertainties in RS information and to test whether the results presented herein can be generalized among crop models with different levels of complexity and across distinct field crops.

## Introduction

According to household surveys, more than 13 million people have limited access to food in South Africa (Bvenura and Afolayan 2015). Drought, caused by large anomalies in annual and interannual precipitation, is the major yield-limiting factor in the rainfed cropping systems of South Africa (Vogel and van Zyl 2016). Most of the maize-growing areas in South Africa are rainfed (Schultz 2006) with a water demand between 450 and 600 mm depending on various climatic and soil variables (du Plessis 2003).

Management decisions, such as the selection of sowing dates, can considerably affect a summer crop’s yield in semiarid regions (Aguirrezábal et al. 2009). The sowing date can directly influence crop yield and yield quality, mainly by changing the temporal patterns of temperature and soil moisture experienced by crops during the growing period (Marteau et al. 2011; Srivastava et al. 2016). Shifting the maize sowing date in the dry regions of South Africa by only 15 days could increase crop yield by 10% (Abraha and Savage 2006). Early sowing dates allow maize to avoid high temperatures and terminal drought during the grain-filling period. On the other hand, late and medium sowing dates can increase the crop growth rate during the early growth period, leading to faster development in leaf area and increasing the plant’s ability to intercept solar radiation (Cirilo and Andrade 1994a, b). The effects of management practices, such as the effect of the optimized sowing date on crop yield, are generally assessed by conducting field experiments (Chen et al. 2011). However, carrying out field experiments is expensive and time-consuming, and the results are limited by the environmental conditions at the study location (Rinaldi 2001; Heng et al. 2007). Hence, testing the suitability of management practices such as optimized sowing dates and cultivar selection is a challenging issue at larger scales (Therond et al. 2011).

Crop growth models have been developed to improve our understanding of these processes and to upscale the effects of management strategies on crop yields and agricultural production from the field level to larger scales (Gaiser et al. 2010). Therefore, employing tools such as crop models supports enhancing the decision-making process for sustainable maize production under variable climatic conditions in South Africa (Zinyengere et al. 2015). In addition, crop models can be used for projections using climate scenarios or to explore the responses of crop yields to altered farm management practices. Most of the crop models used for large-scale modeling experiments are developed at the field level and require a large amount of input data for parameterization and calibration (Van Bussel et al. 2011). However, high-resolution model input data, including sowing dates, are usually not available at a large scale (Rezaei et al. 2015; Zhao et al. 2015). It is therefore common to use a fixed sowing date in large-scale simulation experiments, but failure to consider the spatiotemporal variability of crop model inputs could introduce large uncertainty into the model outputs (Waha et al. 2015).

Various precipitation-based approaches have been introduced to estimate the sowing dates of crops and to consider the spatiotemporal variability of sowing dates in Africa (Dodd and Jolliffe 2001; Laux et al. 2008). The sowing date is determined when the cumulative precipitation at the beginning of the rainy season reaches a certain threshold in a specific period of time (Tachie-Obeng et al. 2013; Waongo et al. 2013). The selection of an appropriate cumulative precipitation threshold, which is related to the temperature pattern and soil characteristics, can be a challenging task. In general, cumulative precipitation-based approaches are relatively reasonable; however, they cannot completely explain African farmers’ decisions since other independent variables, including risk management practices, workload, and variability in soil characteristics, can largely influence their decisions (Waha et al. 2012). In addition, high-quality precipitation data based on a suitable density of climate stations are often limited in Africa (Paeth and Diederich 2011), and this sparsity of data can influence the reliability of rule-based methods for large-scale simulation experiments.

Using remote sensing (RS) to identify the phenology of crops such as maize (Viña et al. 2004) constitutes a promising approach, especially when the aim is to detect the start of the sowing date in semiarid regions with limited ground-based observations (Clinton et al. 2010; Atkinson et al. 2012; Brown et al. 2012). RS information can deliver crop canopy variables such as the leaf area index (LAI), phenology, and evapotranspiration over large spatial areas and thus inform large-scale crop models (Jin et al. 2018). However, the full exploitation of RS information can be hampered by factors such as the presence of clouds and/or dust and topographical effects and by a limited number of observations during the growing season of crops (Zhang et al. 2003; Tucker et al. 2005). In this sense, various RS sensors (MODIS, RapidEye, MERIS, etc.) and indices (LAI, NDVI, EVI, WDRVI, etc.) have been used to detect phenological dynamics to partly overcome these limitations (Jeganathan et al. 2010; Pan et al. 2012; Siachalou et al. 2015). Unlike the flowering stage, which can be related to the maximum LAI (Tagliapietra et al. 2018), estimating the sowing date is more challenging because of the difference between the sowing date and the time when the reflections from green leaves are detectable by the sensors. This time gap is generally addressed by assuming a fixed number of days between sowing and emergence (Dimou et al. 2018), which can introduce considerable uncertainty to the estimated sowing date. The scale mismatch between the low-resolution climate and soil data (particularly in Africa) used by crop models and the finer resolution of RS observations is another limitation of using RS information for large-scale crop modeling experiments (Doraiswamy et al. 2005). In summary, RS information and precipitation data are widely used to independently estimate the sowing date. However, to date, the differences in the sowing dates estimated by different methods have not been quantified.

At present, little is known about the sensitivity of crop model outputs to (i) changes in the fixed sowing date, (ii) variations in the cumulative precipitation threshold, and (iii) the selected time gap between sowing and emergence in RS-derived sowing estimations. In this context, the objectives of the current study are to perform a systematic sensitivity analysis to compare the long-term sowing date estimates based on the MODIS phenology product at the native resolution with those derived with a precipitation rule and to assess the impact of distinct sowing date estimates on the rainfed maize yield simulated for South Africa.

## Materials and methods

### Study area

**Fig. 1:**
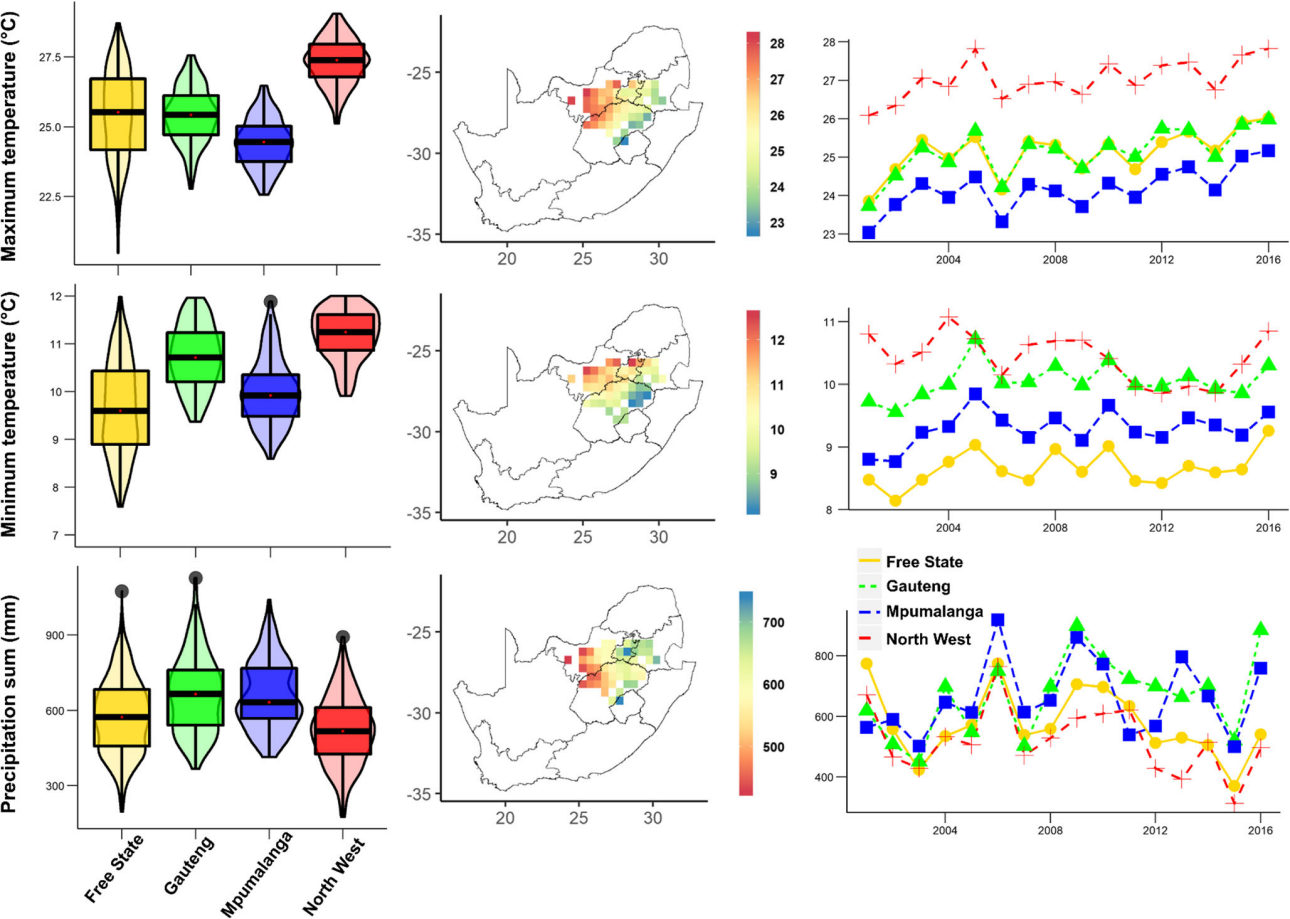
Violin boxplots (**a**), spatial pattern (**b**), and interannual variability (**c**) of the annual precipitation sum and the mean daily maximum and mean daily minimum temperatures in the period 2001–2016 across maize growing grids in Free State, Gauteng, Mpumalanga, and North West Provinces, South Africa. The boxes are related to the 25th and 75th percentiles; the whiskers extend to 1.5 times the interquartile range, and the horizontal line indicates the median. No overlapping of the notches among boxes offers evidence of statistically significant differences among their medians. Black and red dots reflect outliers and means, respectively

This analysis was performed for the provinces of Free State, Gauteng, Mpumalanga, and North West (Fig. 1), collectively covering 329,318 km^2^ and producing approximately 85% of the maize harvest in South Africa (DAFF 2019). Rainfed farming is the most common cultivation pattern of maize within these provinces. An analysis of climate data for the period 2001–2016 showed that maize-growing areas received, on average, 520 mm (North West) to 665 mm (Gauteng) of annual precipitation (Weedon et al. 2014). North West Province has experienced a remarkably higher mean daily maximum temperature (27.1 °C) than the other studied provinces (24.2 °C to 25.2 °C). The mean daily minimum temperature recorded in the provinces varied by 1.8 °C, with Free State experiencing the lowest minimum temperature (8.7 °C) during the study period (Fig. 1).

### General data analysis workflow

**Fig. 2:**
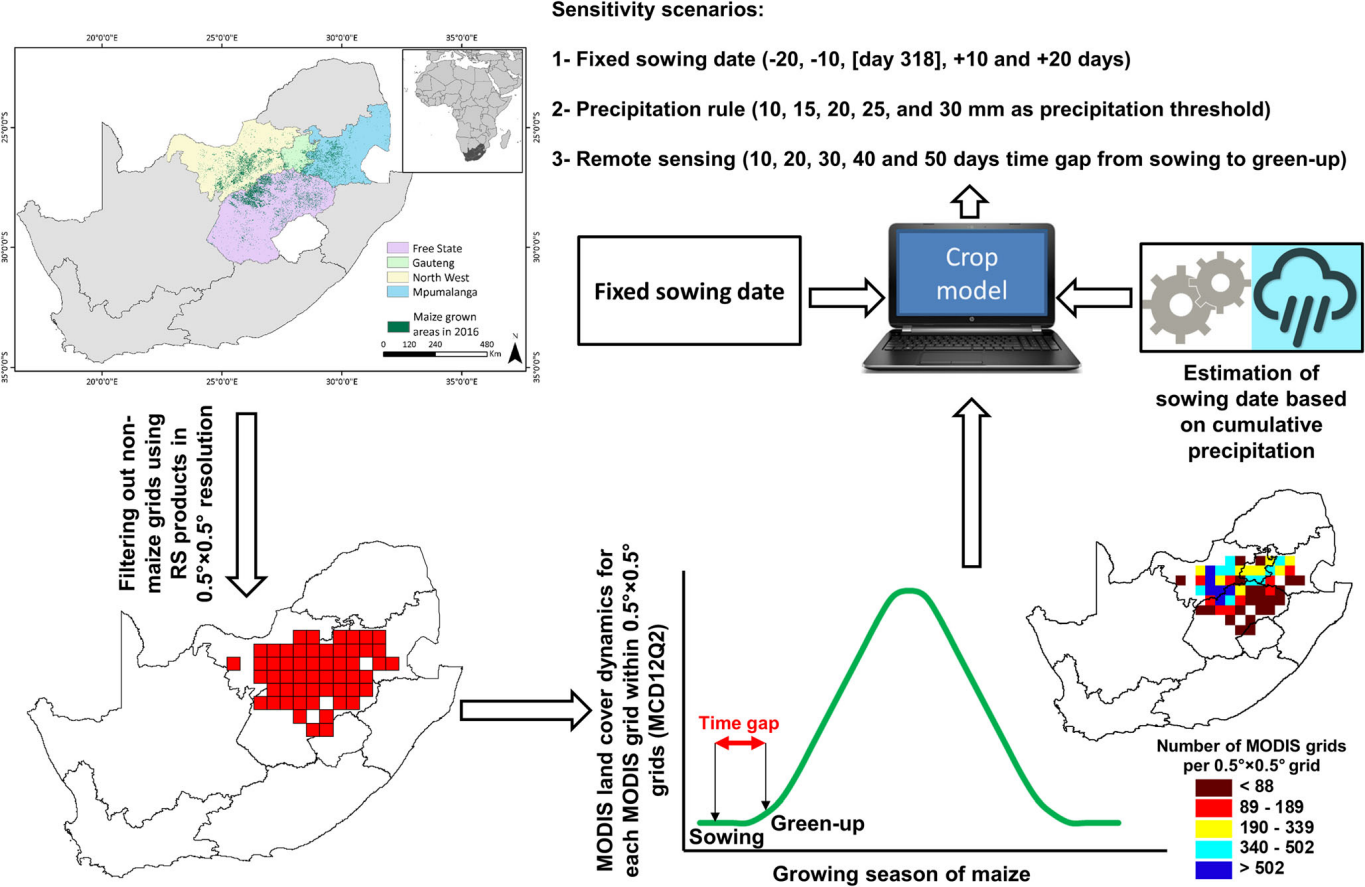
Study region and the workflow for processing the remote sensing information used to inform the crop model to analyze the effects of different sowing date estimation methods on crop model results

Analyzing the sensitivity of model outputs to sowing date estimation methods involved various steps, including data preparation, RS-based data extraction, model setup, and model output analysis (Fig. 2). In the first step, non-maize grids were filtered out across the study areas in South Africa using a maize crop mask. Then, curves of vegetation indices were extracted for each of the remaining grid cells from the time series of the MODIS global vegetation phenology (MCD12Q2) product (Xiao et al. 2013) for the period 2001–2016. Due to the lack of high-resolution climate and soil data as model inputs, the green-up dates extracted from MODIS pixels (500-m resolution) were aggregated to 0.5° × 0.5° grids, consistent with the spatial resolution of available climate and soil data. Five sensitivity scenarios were defined for estimates of the fixed sowing date, precipitation rule-based sowing date, and RS-based sowing date (Fig. 2). The thresholds of cumulative precipitation were defined as 10, 15, 20, 25, and 30 mm. Fixed periods of 10, 20, 30, 40, and 50 days were subtracted from the detected green-up dates as sensitivity scenarios to account for the time gap between sowing and green-up (emergence of leaves), which is when plants become visible to satellites. The fixed sowing date also varied in the range between − 20 and + 20 days relative to the standard sowing date.

The crop model was executed using the sowing dates estimated by using the different methods and thresholds. Next, the spatial and temporal patterns of the estimated sowing dates and simulated yields using different methods were analyzed. Significant differences in the sowing dates and simulated yields obtained using different sowing date estimation methods were tested using ANOVA followed by a Tukey test (HSD) at *P* = 0.05. Anomalies of the simulated yield with different sowing estimation methods were compared with the anomalies of the observed yield in the period from 2003 to 2015.

### Processing of climate, soil, and management data

The WATCH Forcing climate dataset, which was developed using ERA-Interim reanalysis data (Weedon et al. 2014), was used as a climate input for the crop model. This dataset includes daily and 3-hourly values of temperature, precipitation, radiation, and wind speed at a global scale (0.5° × 0.5°) for the period 1979–2016 (Weedon et al. 2014; Müller Schmied et al. 2016). The maximum and minimum values of the 3-hourly temperature data were calculated and assumed to represent the daily maximum and minimum temperatures required by the crop model.

Both physical (saturation, field capacity, wilting point) and chemical (total nitrogen density) soil characteristics with a spatial resolution of 0.5° × 0.5° were provided by the ISRIC-WISE dataset and the Global Gridded Surfaces of Selected Soil Characteristics, respectively (Batjes 2012). Soil depth information was obtained from the FAO soil depth dataset and restricted to 1 m to be compatible with the other soil information (Batjes 1997).

The long-term mean reference sowing dates of rainfed maize in South Africa was obtained from the MIRCA2000 dataset (Portmann et al. 2010). These sowing dates were used for simulations with fixed sowing dates and to define a time window of potential sowing dates for the precipitation rule-based sowing date estimations. Since there is no large-scale phenological database of rainfed maize for South Africa, the long-term mean harvest date was derived from MIRCA2000, and the temperature sum from sowing to harvest was calculated under the assumption that 60% of the temperature sum contributed to the vegetative phase while the remaining 40% contributed to the reproductive phase (van Bussel et al. 2015). The nitrogen fertilizer application rate of maize in South Africa was obtained from a global dataset of fertilizer application rates (Potter et al. 2010).

### Processing of RS information

As a primary data source of RS-based phenology estimates, the MODIS MCD12Q2 Version 6 product was used, as this product offers a validated dataset providing high-quality and consistent outputs, such as green-up, peak, and maturity stage, which are applicable for studies at regional to global scales. In this product, the seasonal dynamics of vegetation are derived using time series of the 2-band enhanced vegetation index (EVI2), which in turn is based on the MODIS nadir bidirectional reflectance distribution function (BRDF)-adjusted reflectance (NBAR) at a spatial resolution of 500 m (Friedl et al. 2019). The dataset was preprocessed using quality masks to exclude low-quality pixels (Stanimirova et al. 2019).

The estimated start of the season, “Greenup_1” (the date when EVI2 exceeded 15% of the EVI2 segment amplitude during the first cycle), was converted to the DOY and extracted for only maize-grown areas using crop distribution information to reduce contamination from neighboring land use classes and to decrease the impact of mixed pixels. The “Greenup_2” product, which accounts for the second growing period, was excluded after the initial test, as it did not provide data for maize-growing areas. The crop layers for the provinces of Free State, North West, and Mpumalanga were obtained from the South African Department of Agriculture, Forestry and Fisheries (DAFF 2017); these maps were generated based on the classification of SPOT imagery. For Gauteng Province, crop information was provided by the Sen2-Agri project (Defourny et al. 2019) based on Sentinel-2 information for 2016. When available, crop information from several years was considered for the analysis.

### Sowing date estimation based on daily precipitation

The sum of daily precipitation was accumulated (in scenarios ranging from 10 to 30 mm) in moving 7-day intervals during the potential sowing period to determine the sowing date. The start of the potential sowing period was defined as the period between the first day of the month provided by MIRCA2000 as the sowing month and the following 50 continuous days. The first day of the first 7-day period with a precipitation sum ranging from 10 to 30 mm (with an interval of 5 mm) was defined as the day of sowing (Srivastava et al. 2016). The last day of the potential sowing period was selected as the sowing date if each 7-day precipitation sum in the potential sowing period was smaller than the predefined threshold.

### Crop model description

SIMPLACE (Scientific Impact assessment and Modeling Platform for Advanced Crop and Ecosystem management) is a modeling framework based on the concept of encapsulating the solution of a modeling problem in discrete, replaceable, and interchangeable software units called Sim-components or sub-models (Rezaei et al. 2015). A specific combination of submodels within the modeling framework is called a model solution. The SIMPLACE < LINTUL-5,Heat > solution employed in this study combines the LINTUL-5 crop model (Wolf 2012) with a module implemented in the SIMPLACE platform to quantify the effect of heat stress on grain yield (Rezaei et al. 2015). The yield-limiting factors considered in the crop model were drought, heat, and nitrogen stress. Due to the lack of experimental data for the model calibration, we used the SIMPLACE model parameters used in a preceding study to simulate the maize yield across Africa (Rezaei and Gaiser 2017) (Table 1). The drought stress reduction factor is based on the ratio between actual and potential transpiration and influences leaf area expansion, root growth, biomass accumulation, and the partitioning of photosynthetic assimilates (Wolf 2012).

**Table 1:** Crop parameters of SIMPLACE < LINTUL-5,Heat > used in the current study

Parameter	Description	Unit	Value
TSUM1	Temperature sum from emergence to anthesis	°C day^−1^	1085–1712
TSUM2	Temperature sum from anthesis to maturity	°C day^−1^	723–1141
RUE-0.0	Radiation use efficiency at development stage 0*	g MJ^−1^ m^−2^	3.0
RUE-1.50	Radiation use efficiency at development stage 1.50	g MJ^−1^ m^−2^	3.0
RUE-2.0	Radiation use efficiency at development stage 2.0	g MJ^−1^ m^−2^	2.4
ROOTDM	Maximum rooting depth	m	1
ROOTDI	Initial rooting depth	m	0.1
RRDMAX	Maximum rate of increase in rooting depth	m	0.012
TDWI	Initial total crop dry weight	kg ha^−1^	5
HSTCritical	Critical temperature threshold which grain yield start to damage	°C	35
RTNMINS	Fraction of soil mineral N coming available per day	-	0.005

### Comparison of estimated sowing dates and simulated yields

We first pooled the sowing date estimates for grids and years assigned to the studied provinces to compute the summary statistics (mean, median, and quantiles) obtained from different sowing date estimation methods and sensitivity scenarios. Outliers were defined based on the distance from the interquartile range (Krzywinski and Altman 2014). Then, we calculated the annual anomalies of the estimated sowing dates and simulated yields across the years to analyze the differences caused by the various sowing estimation methods and scenarios on the interannual yield variability. Simulated yields were compared with the yields of rainfed yellow maize available at the field level for the period from 2003 to 2015 (DAFF 2019). The field-scale observations were aggregated to a resolution of 0.5° × 0.5°, and the yield anomaly was calculated for the grids (29 grids) that had observations for the entire period.

### Statistical analysis

Differences in estimated sowing dates and simulated yields were evaluated using ANOVA (aov function in R) followed by a Tukey test (HSD) at *P* = 0.05 employing the agricolae package (De Mendiburu and Simon 2015) in R. The familywise 95% confidence intervals indicate significant multiple (pairwise) comparisons of means among the sowing estimation methods.

## Results and discussion

### Differences in sowing dates estimated using RS and the precipitation rule

**Fig. 3:**
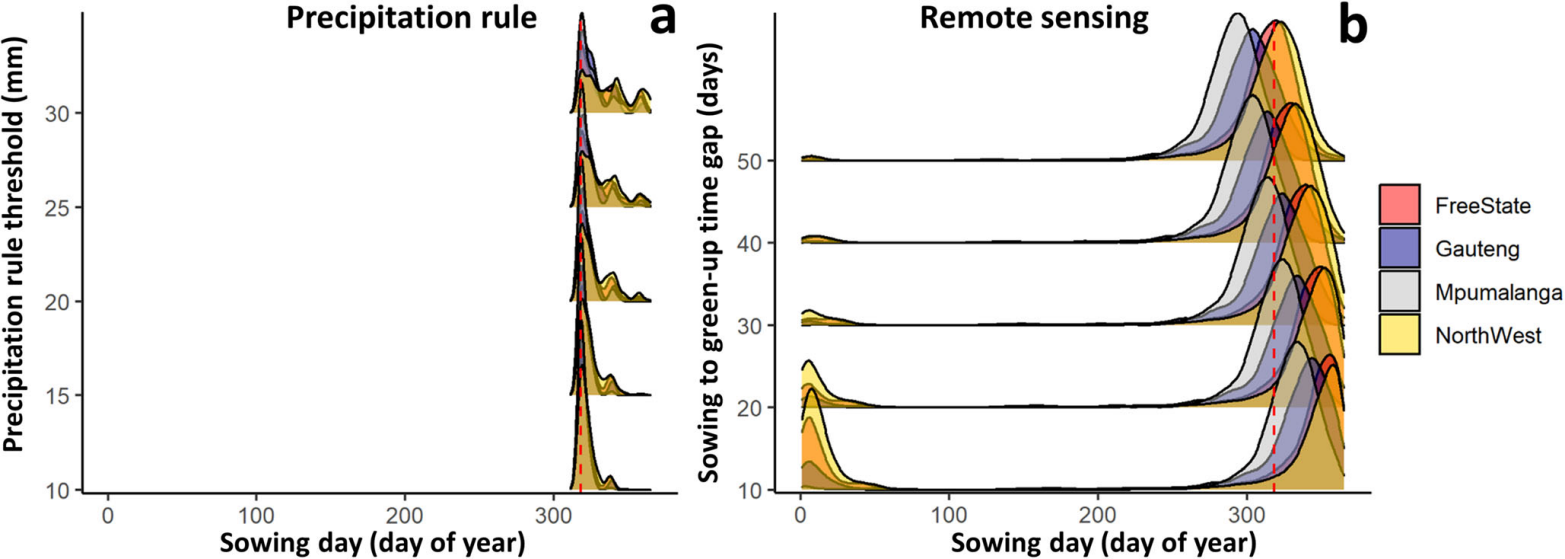
Density ridgeline of the estimated sowing dates of maize using the RS and precipitation rule sensitivity scenarios in the period 2001–2016 across maize growing grids in Free State, Gauteng, Mpumalanga, and North West Provinces, South Africa. The red line indicates a fixed sowing date

The sowing dates were remarkably different among the estimation methods, thresholds, and provinces (Fig. 3). Low precipitation thresholds resulted in relatively similar sowing dates for all studied provinces at day 320 (Fig. 3a), while the estimated sowing date differed among the studied provinces when using high precipitation thresholds. The distribution of sowing dates estimated by using the precipitation rule changed from unimodal (one peak) to multimodal (multiple peaks) from day 320 to day 340 in North West and Free State (Fig. 3a). There is a remarkable difference in the estimated sowing dates between the hot and dry provinces of North West and Free State and the other provinces (Fig. 3b). The earliest RS-derived sowing dates were obtained for Mpumalanga (290–326) (Fig. 3b), and the RS-based sowing dates for North West and Free State were 11 days earlier than the sowing dates estimated for the other provinces (Fig. 3b).

The use of RS information remarkably increased the variability of the estimated sowing dates compared to those estimated with the precipitation rule. Using the precipitation rule resulted in sowing windows spanning 21–46 days depending on the specific precipitation threshold (Fig. 3). This indicates relatively small interannual variability at the beginning of the rainy season in South Africa. However, the actual sowing date window can be much wider because, in addition to climatic factors, other management factors can affect the choice of sowing date (Srivastava et al. 2016). For instance, the optimum temperature range, land preparation, and availability of labor can impact the timing of sowing, particularly for smallholder farmers in South Africa (Waddington et al. 1991). A previous study showed that the sowing date of maize can range from October to January in Free State Province (Moeletsi 2017). Potentially, RS observations can better capture the variability in sowing dates caused by these differences in the onsets of rainfall in cropping systems and socioeconomic limitations (Kasampalis et al. 2018). Using fixed sowing dates or using sowing dates based on the precipitation rule did not account for the wide range of cultivars (short-, medium-, and long-season cultivars) grown in South Africa, which may have different optimum sowing dates (Moeletsi et al. 2013). The effectiveness of using RS information in tracking the development stage was confirmed in previous studies for different crops grown in various environments (Zhang et al. 2003; Viña et al. 2004).

### Effects of differences in the estimated sowing dates on the simulated yield

**Fig. 4:**
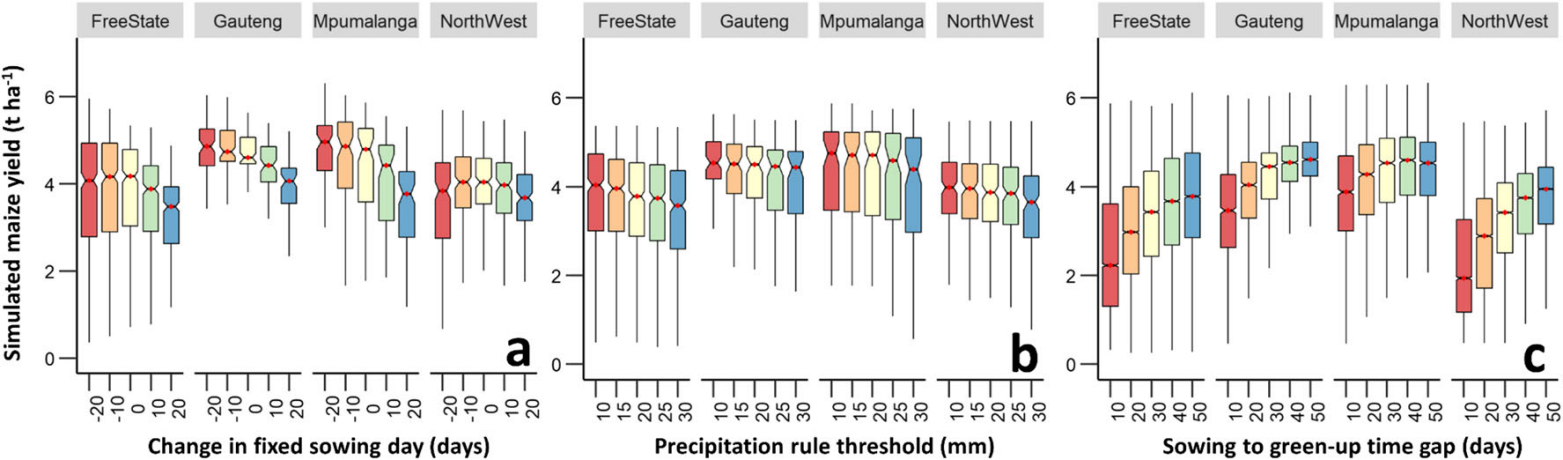
Boxplots of simulated yields of maize using sensitivity scenarios based on fixed sowing dates (**a**), sowing dates estimated by the precipitation rule (**b**), and RS-based sowing dates (**c**) in the period 2001–2016 across maize growing grids in Free State, Gauteng, Mpumalanga, and North West Provinces, South Africa. The boxes refer to the 25th and 75th percentiles; the whiskers extend to 1.5 times the interquartile range, and the horizontal line indicates the median, where no overlapping of the notches among boxes offers evidence of statistically significant differences among their medians. The red dots represent the mean values

**Fig. 5:**
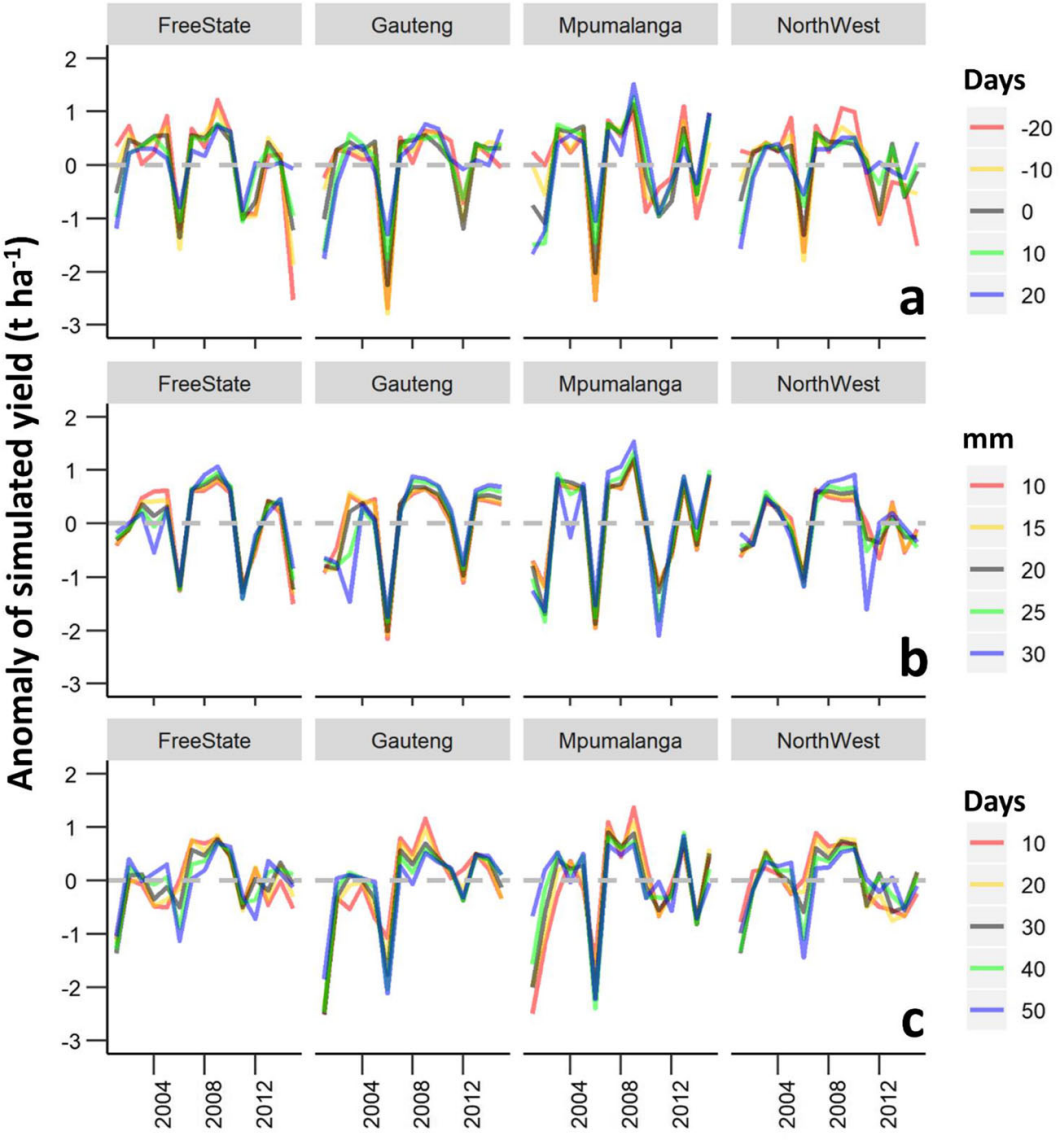
Anomaly of the simulated yield of maize using sensitivity scenarios based on fixed sowing dates (**a**), sowing dates estimated by the precipitation rule (**b**), and RS-based sowing dates (**c**) in the period 2001–2016 across maize growing grids in Free State, Gauteng, Mpumalanga, and North West Provinces, South Africa

**Fig. 6:**
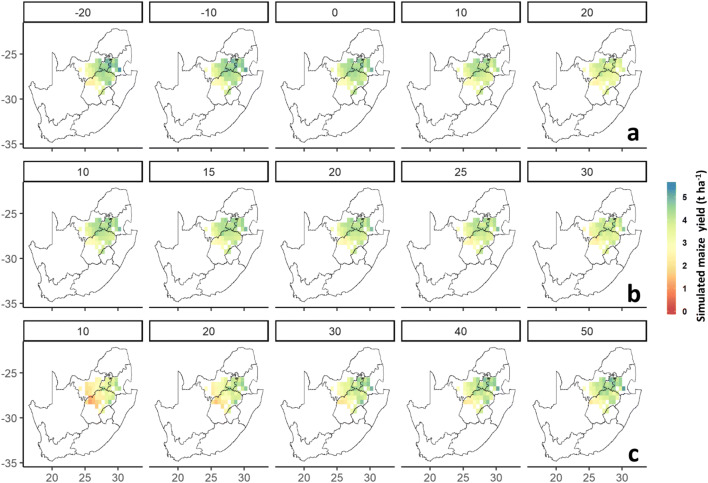
Spatial pattern of the mean yield of maize simulated using sensitivity scenarios based on fixed sowing dates (**a**), sowing dates estimated by the precipitation rule (**b**), and RS-based sowing dates (**c**) in the period 2001–2016 across maize growing grids in Free State, Gauteng, Mpumalanga, and North West Provinces, South Africa

The simulated maize yield calculated as the mean across grids and years was in the range between 2.51 and 4.04 t ha^−1^ (Fig. 4). The mean simulated yield in Mpumalanga and Gauteng was 0.6 t ha^−1^ higher than that in North West and Free State. Early sowing dates resulted increases in the mean simulated yields in all studied provinces except North West (Fig. 4a). Increasing the cumulative precipitation rule threshold from 10 to 30 mm reduced the mean simulated yield between 7 and 12% depending on the studied province (Fig. 4b). Expanding the time gap between sowing and green-up to 50 days resulted in a 0.56 to 1.48 t ha^−1^ increase in the simulated yield, particularly in the hot-dry provinces (Fig. 4c). The standard deviation of the simulated yield anomaly was 40% larger for provinces with higher simulated yields, such as Gauteng and Mpumalanga, than for Free State and North West (Fig. 5). In general, early sowing dates, large precipitation thresholds, and large differences between the sowing and green-up dates resulted in larger anomalies of simulated yields (Fig. 5). An analysis of the spatial patterns of the simulated yields showed a decreasing trend of the simulated yield from west (3.0 to 6.0 t ha^−1^) to east (0.3 to 3.0 t ha^−1^), particularly when the RS-based sowing date estimations were employed across the maize-growing areas (Fig. 6). This pattern was less pronounced when using fixed sowing dates and precipitation rule-based sensitivity scenarios (Fig. 6).

**Fig. 7:**
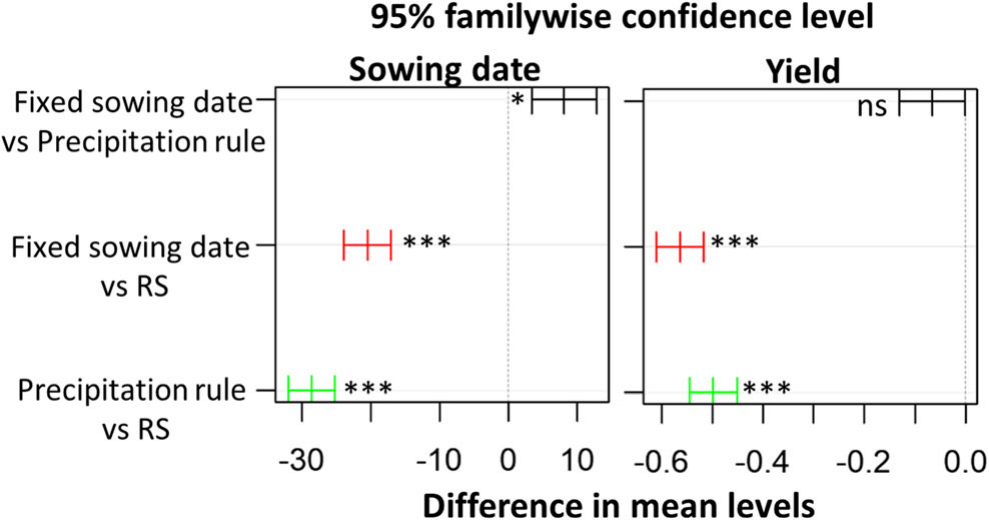
Multiple (pairwise) comparisons among the means of the estimated sowing dates (**a**) and simulated yield (**b**) of maize using RS dates, fixed sowing dates and the dates estimated with the precipitation rule using the Tukey test in the period 2001–2016 across maize growing grids in Free State, Gauteng, Mpumalanga, and North West Provinces, South Africa. ns, nonsignificant; * and *** *p* ≤ 0.001 and 0.05 significance levels, respectively

The results of the Tukey test showed a significant difference among the methodologies for estimating sowing dates (Fig. 7). There was also a significant difference between the simulated yields using the RS-based sowing estimations and the other sowing estimation methods (Fig. 7). However, there was no significant difference in the simulated yield between the fixed date and precipitation rule-based sowing estimations (Fig. 7).

The simulated yield was significantly affected by the selected method and the sensitivity scenario used to estimate the sowing date (Figs. 3 and 4). These differences highlight the importance of selecting a robust and suitable sowing date estimation method and threshold in large-scale simulation experiments, particularly when ground-based observations are not available (Ogutu et al. 2018). The maize sowing dates in the semiarid regions of Africa are generally tied to the start of the rainy season (Sacks et al. 2010). However, the assumption that all farmers (particularly smallholder farmers) in certain provinces decide to sow at the optimum time (e.g., at the fixed sowing date given in large-scale modeling experiments) is an oversimplification of real conditions (Srivastava et al. 2016), since the farmers in large growing areas of South Africa have different levels of agronomic knowledge (Muzangwa et al. 2017) and different capacities to deal with labor peaks. In addition, farmers cannot know at the beginning of the growing season how the climatic conditions will subsequently develop. Therefore, it is, in principle, impossible to determine the optimal sowing date in real time.

**Fig. 8:**
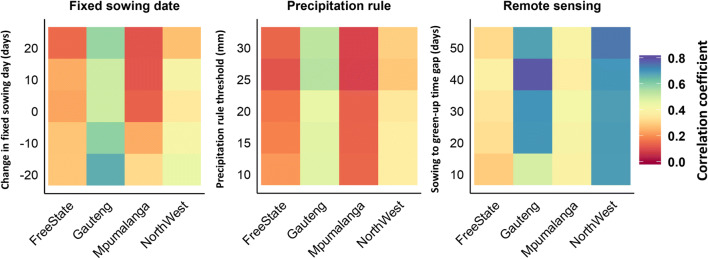
Correlation coefficients between the anomaly of simulated yield using sensitivity scenarios based on RS-based sowing dates, fixed sowing dates and the dates estimated with the precipitation rule and the observed yield in the period 2003–2015 across maize growing grids in Free State, Gauteng, Mpumalanga, and North West Provinces, South Africa

The anomaly of the simulated yield using the RS estimation method indicated a stronger (*r* = 0.30 to 0.79) correlation with the anomaly of the observed yield, particularly in Gauteng and North West, than the correlations between the simulated and observed yield anomalies when using a fixed sowing date (*r* = 0.12 to 0.66) or a precipitation rule-based sowing date (*r* = 0.10 to 0.54) (Fig. 8).

The RS-based sowing date estimations resulted in a better representation of the spatiotemporal variability of yield, at least for Gauteng and Mpumalanga Provinces (Fig. 6). Using the fixed sowing or precipitation rule-based sowing date estimation methods resulted in a loss of information regarding the spatial yield pattern (Fig. 6). The failure to detect the variability of the observed yield using a fixed sowing date can be related to the use of low-resolution climate and soil inputs for our large-scale simulations. Using the model inputs at a low spatial resolution (0.5° × 0.5° in our case) ignored the heterogeneity of precipitation, temperature, soil characteristics, and agronomic management (such as the sowing date) within the grids. Such variability can be partly counterbalanced by using RS- derived information. The use of RS products for estimating the sowing date could be a better approach than using a fixed sowing date. Using a fixed sowing date does not account for the interannual precipitation pattern and ignores the spatial pattern of sowing dates, assuming that all farmers sow crops at the same time (Wolf et al. 2015). Using the precipitation rule-based sowing estimation approach always depends on the selected start date of the potential sowing period, which in turn determines how the period with continuous precipitation events is found.

Based on the results of our sensitivity analysis, the thresholds leading to earlier sowing date estimations resulted in higher simulated yields in most of the provinces. This result may be caused by the fact that the time between sowing and emergence was not impacted by soil moisture in the topsoil in the crop model used in this study. In reality, emergence would be delayed if the topsoil is very dry (Blackshaw 1991). The results of this sensitivity analysis may therefore change when using other crop models accounting for the relationship between soil moisture and the duration between sowing and emergence.

### Limitations of the data and methods used in this study

Through this study, we improved our understanding of the impact of distinct sowing date estimation methods and the selection of appropriate thresholds (for each method) on simulated maize yields. However, we also need to acknowledge some shortcomings of our input data and of the methods presented here. We were not able to conduct an extensive model calibration due to a lack of time series of experimental data for crop phenology. We employed yields observed at the field scale in the period 2003–2015 for a comparison to yield anomalies simulated using distinct methods and thresholds for detecting the sowing date. Therefore, we can quantify the sensitivity of yields to the choice of the sowing date estimation method, but we cannot determine which method works most precisely (in the yield simulation) for the study period. However, this may not change the merit of the present study, as an extensive calibration would improve only the fit of the simulated yields to the observed yields but not the difference among the sowing estimation methodologies. In addition, improving the model itself was not the objective of this study.

We also did not have access to information about the phenological characteristics (differences in thermal requirements) and the proportions of local and commercial maize cultivars in the specifically studied provinces of South Africa. Additionally, maize maps were only available for a few study years, which implies the assumption for dataless years that maize was sown at the same place with no rotation or fallow periods in between. The crop model also did not account for biotic stress factors such as pests or diseases of maize in the study region, which can influence the selected sowing dates by farmers and thus affect crop yield (Martin and Shepherd 2009; Bennett et al. 2012).

Finally, the inconsistency between the spatial resolutions of remote sensing information (high resolution) and those of model inputs such as climate and soil (low resolution) can reduce the improvement achieved by using RS information due to the required aggregation of RS information to a coarser resolution. We also did not have information about the spatial extent of irrigated maize, which may have a completely different sowing window from that of rainfed maize.

## Conclusions

We conclude that the choice of method for estimating the sowing date and the selected threshold causes considerable differences in estimated sowing dates and in crop yields simulated by using these sowing dates as inputs. To improve the accuracy of yield simulations, it is therefore essential to reduce the uncertainty in the spatiotemporal pattern of sowing dates. Using RS- derived sowing date estimations can result in a better representation of the variability of crop yield compared to using a fixed sowing date. Considerable spatiotemporal variability was observed in the estimated sowing date using RS-derived information, particularly in the dry provinces. Improving the reliability of RS sources (e.g., through higher temporal and spatial resolutions and extensive RS product validation) and of techniques for extracting phenological metrics could help to reduce the number of outliers. The sensitivity of the model results to the sowing date estimations was substantially larger in drier and warmer provinces owing to the higher variability of the exposure of crops to heat and drought caused by varying sowing dates.
